# Pathological mutations differentially affect the self-assembly and polymerisation of the innate immune system signalling adaptor molecule MyD88

**DOI:** 10.1186/s12915-018-0611-7

**Published:** 2018-12-24

**Authors:** Ailís O’Carroll, Brieuc Chauvin, James W. P. Brown, Ava Meagher, Joanne Coyle, Jurgen Schill, Akshay Bhumkhar, Dominic J. B. Hunter, Thomas Ve, Bostjan Kobe, Emma Sierecki, Yann Gambin

**Affiliations:** 1 EMBL Australia Node in Single Molecule Science, University of New South Wales, Kensington, NSW 2052 Australia; 2 Institute for Molecular Bioscience, University of Queensland, QLD, Brisbane, 4072 Australia; 3 Institute for Glycomics, Griffith University, QLD, Southport, 4222 Australia; 4 School of Chemistry and Molecular Biosciences, and Australian Infectious Diseases Research Centre, University of Queensland, QLD, Brisbane, 4072 Australia

**Keywords:** MyD88, Polymerisation, Prion-like, Disease-associate point mutations, Single molecule, Eukaryotic cell-free expression

## Abstract

**Background:**

Higher-order self-assembly of proteins, or “prion-like” polymerisation, is now emerging as a simple and robust mechanism for signal amplification, in particular within the innate immune system, where the recognition of pathogens or danger-associated molecular patterns needs to trigger a strong, binary response within cells. MyD88, an important adaptor protein downstream of TLRs, is one of the most recent candidates for involvement in signalling by higher order self-assembly. In this new light, we set out to re-interpret the role of polymerisation in MyD88-related diseases and study the impact of disease-associated point mutations L93P, R196C, and L252P/L265P at the molecular level.

**Results:**

We first developed new in vitro strategies to characterise the behaviour of polymerising, full-length MyD88 at physiological levels. To this end, we used single-molecule fluorescence fluctuation spectroscopy coupled to a eukaryotic cell-free protein expression system. We were then able to explore the polymerisation propensity of full-length MyD88, at low protein concentration and without purification, and compare it to the behaviours of the isolated TIR domain and death domain that have been shown to have self-assembly properties on their own. These experiments demonstrate that the presence of both domains is required to cooperatively lead to efficient polymerisation of the protein. We then characterised three pathological mutants of MyD88.

**Conclusion:**

We discovered that all mutations block the ability of MyD88 to polymerise fully. Interestingly, we show that, in contrast to L93P and R196C, L252P is a gain-of-function mutation, which allows the MyD88 mutant to form extremely stable oligomers, even at low nanomolar concentrations. Thus, our results shed new light on the digital “all-or-none” responses by the myddosomes and the behaviour of the oncogenic mutations of MyD88.

## Background

In the innate immune system, dedicated germline encoded receptors known as pattern recognition receptors (PRRs) recognise pathogens from all major classes of invading microorganisms, as well as other endogenous danger-associated molecular patterns. The Toll-like receptors (TLRs) are a major family of PRRs whose signalling pathways culminate in the activation of transcription factors that mediate innate immune responses and therefore have a crucial regulatory function in maintaining health and eradicating disease [1]. TLRs recruit different combinations of the four key adaptor proteins (TRIF, TRAM, Mal and MyD88) [2]. MyD88 is the least polymorphic adaptor and has evolved under purifying selection, confirming its role as an essential and non-redundant protein in host survival [3]. This implies a crucial role in signalling. Patients with MyD88 mutations such as the L93P and R196 point mutations present a primary immunodeficiency syndrome characterised by greater susceptibility to pyogenic Gram-positive bacterial conditions [4–6], which often result in life-threatening infections. Somatic mutations in MyD88 have also been found which contribute to human malignancies for both chronic lymphocytic leukaemia and more commonly diffuse large B cell lymphoma [7]. In particular, the L252P point mutation (also referred to as L265P in previous studies) was discovered to be driving the promotion of malignant cell survival in many lymphoma patients [4].

MyD88 is recruited to TLR4 through another adaptor protein, Mal. Upon receptor oligomerisation [2], Mal acts as a nucleation platform for the downstream recruitment of MyD88 through homotypic interactions between their respective Toll-interleukin-1 receptor (TIR) domains [8]. MyD88 also possesses a death domain (DD) (amino acids 1–110) that binds to and recruits the downstream kinases, IRAK2 and IRAK4 [9]. The DDs of MyD88, IRAK2 and IRAK4 co-assemble into the well-defined “myddosome”. The structure of the Myddosome was solved by crystallography [9–11] and shows a helical organisation of six to eight DD of MyD88, four IRAK2 and four IRAK4 DD molecules [9]. However, as only the DD of MyD88 was used in these studies, the spatial localization of the TIR domain of MyD88 and its role in the myddosome assembly could not be determined. In parallel, recent studies demonstrate the role of the TIR domain in the self-assembly of MyD88. Ve et al. discovered that the recombinant isolated TIR domains of Mal and MyD88 can self-organise into helical filaments at high protein concentrations and have solved the structure of this assembly by cryo-electron microscopy [12]. The TIR filaments of both Mal and MyD88 have self-replicating propensity [12]. Again, these studies were conducted on isolated TIR domains and the impact of the DD is unknown.

Overall, a growing list of “prion-like” polymers have been found within the proteins of the innate immune system, namely RIPK1, RIPK3 [13], mitochondrial antiviral-signalling protein (MAVS), and apoptosis-associated speck-like protein containing caspase recruitment domain (CARD) (ASC) [14–16]. A novel paradigm is emerging, recently termed “signalling by cooperative assembly formation” or SCAF [17–19], whereby the response of the innate immune system is driven through the polymerisation of adaptor proteins, creating a highly non-linear amplification of the signal. Interestingly, the term “prion-like” has been used for the helical assemblies of MAVS and ASC to describe the self-catalysis of polymerisation. In the case of “conventional” prions, the assembly in filaments is linked to a large change in secondary structure, with the creation of stacking β-sheet. For MAVS and ASC, the polymerisation is not seeded and sustained by unfolding of the monomer, and the proteins appear to retain their native folded conformation. Intriguingly, many adaptors with prion-like properties contain two domains, both with self-assembly properties. For example, in the case of ASC, the PYD and CARD domains can both form filaments separately, and the structure of the full-length ASC protein upon polymerisation is still unknown [20, 21]. Similarly, for MyD88, both DD-myddosome and TIR filament structures have been solved but the interplay between the two domains and their contribution to both processes are unknown.

To characterise the contribution of self-association propensities of the full-length MyD88, we used a combination of single-molecule fluorescence microscopy techniques and “in-vitro” protein expression. This enabled us to characterise the behaviour of full-length MyD88 compared to that of the individual domains, at low concentrations and without purification. We observed that the presence of the two domains is necessary to efficiently lead to protein polymerisation. Both domains participate in giving MyD88 its “prion-like” propensity, providing auto-inhibition in the system and ultimately creating a large range of concentrations where the protein is metastable. Using the same system, we studied the effect of three disease-associated point mutations, one mutation in the DD (L93P) and two within the TIR domain (R196C and L252P). All three mutations affect the ability of MyD88 to form full-size polymers. However, in contrast to the two pyogenic bacterial disease-associated point mutants, L93P within the DD and R196C within the TIR domain, the L252P mutant dramatically enhances MyD88 self-association. Indeed, this point mutant forms well-defined and extremely stable oligomers at 40-fold lower concentration than the wild-type protein. Therefore, pathological mutations in MyD88 can lead to both loss and gain of function by modulating the ability of the protein to self-assemble.

## Results

First, it was necessary to look at the self-assembly of full-length MyD88. Because both the TIR domain and the DD have the ability to self-assemble, the contribution of both domains in the behaviour of the full-length protein was of interest. Full-length MyD88 is difficult to express and purify recombinantly in *E. coli*, presumably due to its polymerisation propensity. Here we expressed the proteins in vitro, at controlled low concentrations, and studied their self-association in undisturbed samples using single-molecule counting techniques. More precisely, we used an in vitro translation system derived from *Leishmania tarentolae* [22] (*Leishmania tarentolae* extract, LTE). This eukaryotic system enables the rapid production of proteins (typically within 2 h) and the analysis of protein-protein interactions in a system orthogonal to the human proteome. By controlling the concentration of DNA priming the expression system, we can tune the final expression levels of proteins and co-express proteins at controlled ratios. We have tested this combination on a variety of biological systems and have demonstrated that the flexibility of cell-free protein expression is a great asset to study protein self-assembly [20, 23–26].

To determine the aggregation and oligomerisation propensity of proteins, we have developed diverse “counting” methods based on single-molecule fluorescence techniques. In single-molecule fluorescence spectroscopy, rare protein complexes can be easily detected in a background of monomers and their size can be evaluated by simply counting the number of fluorophores present in each complex. As we demonstrated recently in our study of the prion-like behaviour of ASC, these counting methods are well suited to study the heterogeneous processes of protein oligomerisation and polymerisation [20]. To visualise MyD88 and its mutants, the proteins were expressed as fusions with genetically encoded GFP or mCherry fluorophores and could be measured directly upon expression without further labelling, purification or enrichment steps. The levels of fluorescence obtained provided a direct readout of protein expression levels, after careful calibration with GFP/mCherry protein controls.

**Fig. 1:**
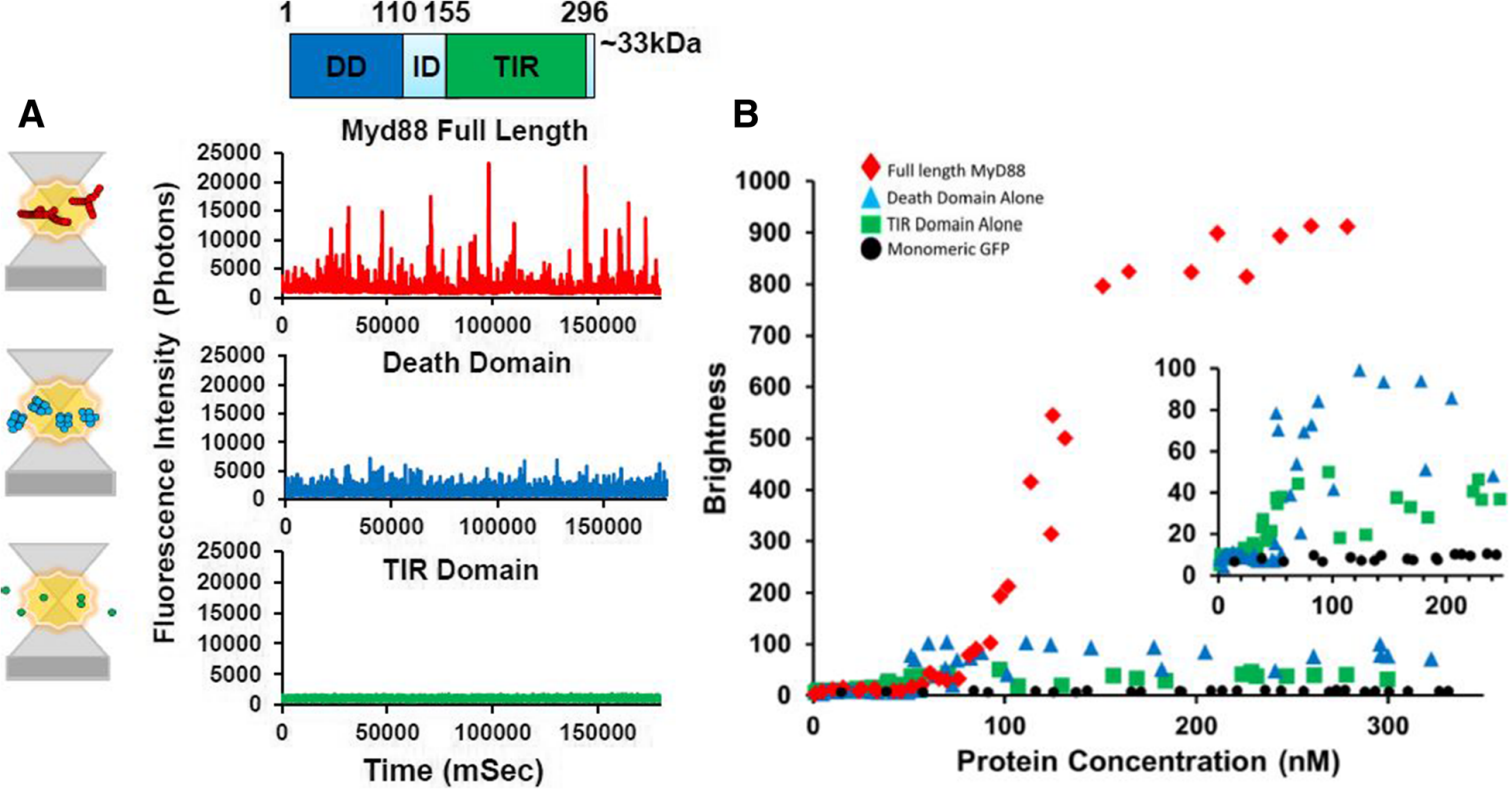
The domains of MyD88 exhibit different oligomerisation propensities, with only the full-length protein forming polymers. **a** Schematic diagram of single-molecule counting experiments, demonstrating the distinction between the oligomeric protein sizes measured, as the green fluorescently tagged protein complexes excited by a 488-nm laser diffuse freely in and out of the focal volume. The schematic diagrams reflect the fluorescence time-traces obtained. The diffusion of an oligomer equates to the same number of fluorophores moving through the confocal volume, creating a burst of fluorescence in the time-trace being directly proportional to the size of the oligomer. For GFP-tagged MyD88 TIR domain, small fluctuations in intensity are recorded around the average fluorescence value, as expected for a low-order oligomer such as a dimer (for example, if 20 proteins are detected simultaneously, the exit/entry of a single protein causes a decrease/increase of signal of only 5%). The GFP-tagged MyD88 DD shows larger bursts of fluorescence correlating with these death domains forming higher order oligomeric complexes. As seen by the fluorescent time traces, N-terminally GFP-tagged full-length MyD88 shows extremely large filamentous polymers of MyD88 diffusing through the confocal volume. **b** The *B* parameter (brightness) correlates with the number of oligomers detected in typical time-traces as a function of protein concentration (nM), for the TIR domain (green), DD (blue) and wild-type full-length MyD88 (red). Protein concentrations range from 0 to 320 nM. Monomeric GFP (black) is included as a control. Inset: expansion of the signals obtained for the individual domains, over a lower concentration range. Fluorescence intensity time traces in **a** are representative traces obtained at > 200 nM protein concentrations. Values in **b** are from approx. 30 dilution experiments with the various protein concentrations and corresponding brightness values obtained plotted

First, constructs containing the TIR domain alone (residues 159–296), death domain alone (residues 1–117, to include crucial part of the intermediate domain (ID)) and full-length MyD88, each fused to GFP at the N-terminus were expressed in LTE. After expression, samples were directly measured on a confocal microscope. A 488-nm laser was focused into the sample, creating a small focal volume, through which proteins could freely diffuse due to Brownian motion. In the range of concentrations used, multiple fluorophores are always present in the focal volume and as proteins constantly exchange in the detection volume, we ultimately interrogate a large number of proteins. Fluctuations of fluorescence intensity were recorded using high-speed single-photon counters; typical fluorescence time-traces obtained are shown in Fig. 1a.

### At low concentrations, both the TIR and death domains are required for efficient polymerisation of MyD88

The fluorescence time-traces obtained for the full-length and separate domains of MyD88 exhibit very distinct characteristics. For TIR domain alone (Fig. 1a, in green), the fluctuations of intensity around the average value are limited (± 500 photons/ms); however, for the death domain (Fig. 1a, in blue), small bursts of intensities (> 1500 photons/ms above background) can be detected. These fluorescence peaks correspond to entries of single protein complexes, increasing the local number of proteins for a brief period of time. The amplitude and duration of the deviations from average are linked to the number of proteins co-diffusing in a single complex and the physical size of the diffusion complex. For full-length MyD88, we observe extremely bright and long-diffusing bursts of fluorescence, as shown in red.

The simplest analysis to quantify the presence of protein complexes is to calculate the average brightness of the diffusing species [20, 27, 28]. The brightness parameter *B* is calculated from the measured intensity values (*I*) as

1 $$ B=\frac{{\left(\mathrm{Standard}\ \mathrm{deviation}\ (I)\right)}^2}{\mathrm{average}\ (I)} $$

The main advantage of this *B* parameter is that it is independent of protein concentrations—in the absence of self-association, the *B* values should be constant as a function of expression levels, and increases of *B* values report on the formation of complexes. Variation of the final levels of protein expression was achieved by using serial dilutions of the priming DNA template in our cell-free expression system, with lower DNA concentrations resulting in lower protein concentrations. For each experiment, fluorescence over time was recorded and the brightness parameter was calculated and plotted as a function of protein concentration. As shown in Fig. 1b, all three constructs display self-association compared to the GFP control. TIR alone forms relatively small oligomers, and the concentration-dependence shows that TIR domains self-assemble at approximately 50 nM. The DD construct forms larger assemblies by itself and the concentration dependence shows a sharp increase into self-assembly at 60 nM. The full-length protein displays much higher brightness values, with the concentration for self-assembly into polymers at approximately 120 nM.

**Fig. 2:**
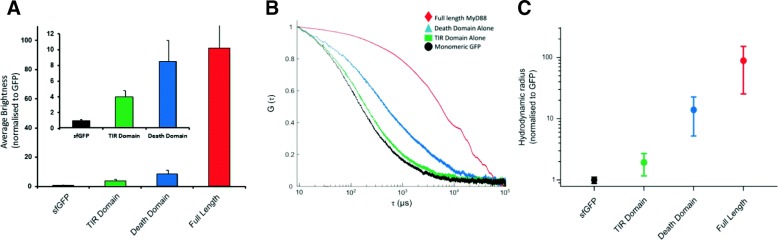
Characterisation of oligomerisation of the TIR and death domains alone and full-length MyD88. **a** Increase of apparent brightnesses of diffusing species. Values of *B* parameters obtained for expressions at protein concentrations > 150 nM were averaged and normalised to the sfGFP monomer control. Clearly, the data show that only full-length MyD88 (red) is capable of forming large complexes, while the TIR domain (green) or the death domain (blue) form smaller oligomers. Inset: Expansion of the values obtained for TIR and death domains compared to sfGFP control. **b** FCS data in solution. Control based on GFP monomer (black). Correlation curves obtained for the TIR domain (green), death domain (blue) and full-length MyD88 (red). A clear shift in diffusion time can be seen between the full-length protein and the separate domains. FCS curves are representative traces from three repeated measurements. **c** Hydrodynamic radius normalised by the sfGFP control (black) calculated for the domains and full-length MyD88, indicating the increase of approximate physical size of the oligomeric species. Values are mean ± SD from three repeat measurements

The average of the brightness values measured above threshold (> 150 nM) can be normalised by the monomeric sfGFP control. As shown in Fig. 2a, the average diffusing complexes formed by TIR and DD are small (approximately four and eight times brighter than monomeric GFP), while the full-length MyD88 has a greatly increased *B* value (100-fold larger). As the samples are heterogeneous, the precise number of proteins in the assemblies cannot be inferred directly using the average brightness value; however, it indicates that the TIR, DD and full-length (FL) MyD88 all form oligomers, with the full-length protein forming much larger species.

To characterise these assemblies in more detail, we performed fluctuation correlation spectroscopy (FCS) experiments (Fig. 2b), which report on the physical size of the diffusing particles. In the case of heterogenous samples with different brightnesses, the larger, brighter species contribute more (to the square of the oligomer size) to the autocorrelation function (*G*(*τ*)) than less bright, monomeric species. Here, a model of a single diffusing species fit the monomeric GFP, TIR, DD and FL data well. Fits were not improved by the addition of further components to the model. The average hydrodynamic radii obtained are plotted in Fig. 2c and normalised to sfGFP. Qualitatively, this data agree with the previous results, with the TIR, DD and FL protein complexes progressively increasing in average size. For the full-length protein, the FCS data show the presence of large species, probably higher order polymers. The average diffusion time is around 100-fold slower (i.e. hydrodynamic radius 100-fold larger) than that of sfGFP alone, suggesting that the higher order species are composed of > 100 monomeric units. Note that the monomeric unit of GFP-fused FL MyD88 would have a radius ~ 1.4× larger than sfGFP.

Comparisons of the brightness values and FCS data obtained confirm that in our hands, the TIR domain species are low-order oligomers (Fig. 2a-c), at concentrations > 50 nM (Fig. 1b). The absence of large events is consistent with the findings by Ve et al. that MyD88 TIR alone does not spontaneously polymerise [12]. Similarly, our data obtained on the separate death domain demonstrates that it too consistently forms small oligomers, with a hydrodynamic radius eight to ten times larger than monomeric GFP. This data complements previous myddosome data that shows assemblies of six to eight MyD88 DDs [29]. At our concentrations (< 300 nM), only full-length MyD88 is able to form very large assemblies, with a hydrodynamic radius 100-fold larger than sfGFP. Interestingly, the two domains seem to cooperate in the formation of higher order structures, and the full-length assemblies are much larger than the sum of the two individual domain oligomers. This cooperativity also appears to delay the self-assembly of the full-length protein, as the self-assembly transition occurs at higher protein concentrations (120 nM vs 50 nM). Overall, our in vitro results are consistent with the recent single molecule fluorescence microscopy studies by Latty et al. [29] demonstrating the formation of both smaller (approximately six MyD88 complexes) and “super” myddosomes at the cell surface. Our single-molecule traces show clearly the presence of anomalously bright events with > 100 proteins co-diffusing simultaneously.

### MyD88 aggregation is a concentration-dependent, self-templated polymerisation event

Figure 1b shows that the aggregation of full-length MyD88 is a concentration-dependent process and reveals a sharp transition in behaviour at around 120 nM.

**Fig. 3:**
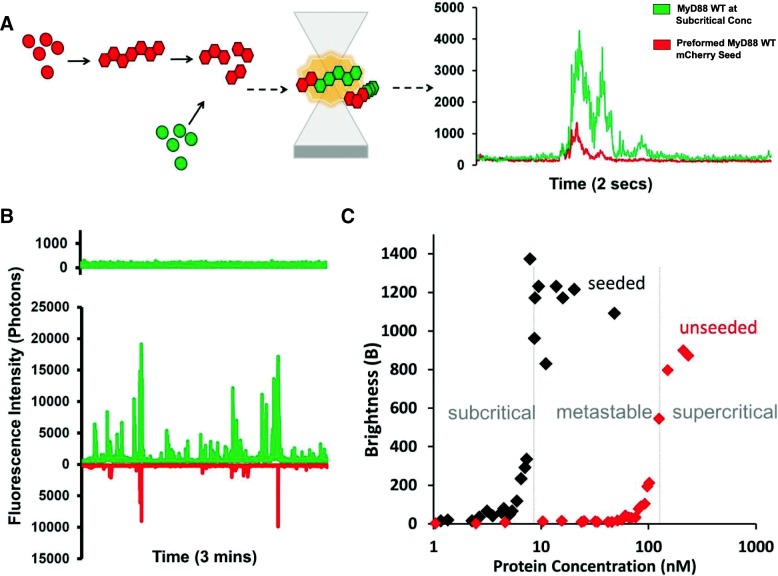
MyD88 polymerises in a concentration-dependent manner and can be self-seeded. **a** Schematic diagram of the principle of two-colour seeding experiments testing the self-replication propensity of full-length MyD88 filaments. Full-length MyD88 is expressed in an mCherry-tagged version above its supercritical concentration to create filaments, which are gently spun and washed, then sonicated to increase the number of fragments. These “seeds” are then mixed in a sample expressing GFP-tagged full-length MyD88 at sub-critical concentrations. **b** Example of fluorescence time trace for MyD88 at 10 nM concentration. Unseeded sample demonstrating a monomeric time trace profile (above) with the seeded sample (below) showing polymerisation of GFP-MyD88 upon the addition of MyD88 “seeds”. **c** The *B* parameter (brightness) correlating with number of oligomers detected in typical time-traces as a function of protein concentration (nM), with and without “seeds” introduced. The subcritical, supercritical and “meta-stable” zones are labelled. Values are from approx. 50 repeated dilution experiments with the various protein concentrations and corresponding brightness values obtained plotted

To confirm that MyD88 filaments can template the conversion from a soluble, monomeric species to a fibrillar form, we used a two-colour seeding assay (Fig. 3a). Briefly, full-length MyD88 tagged with mCherry was expressed at a concentration at which filaments readily form (~ 250 nM). Filaments were enriched by gentle spinning and sonication before being added to solutions containing MyD88 tagged with GFP expressed across a range of concentrations, as previously described. Self-templating was then investigated by two-colour single-particle coincidence spectroscopy. For these experiments, two lasers (488 nm and 546 nm) are focused on the same focal volume, allowing both mCherry and GFP-tagged proteins to be detected at the same time. A typical fluorescence time-trace demonstrating two colour-coincidence experiments with MyD88 is shown in Fig. 3a. The initial expression of GFP-tagged MyD88 is at subcritical concentrations. In the absence of mCherry-tagged MyD88 sonicated filaments (“seeds”), the GFP trace shows little fluctuation confirming that MyD88 is monomeric at this concentration. The mCherry-tagged MyD88 seeds were then added to the mixture and were detected in the mCherry channel. If GFP is recruited to the mCherry seeds, the presence of coincident bursts of fluorescence in both channels results. Indeed, within 20 s, large bursts of fluorescence in the GFP channel were seen and found predominantly to coincide with the presence of mCherry peaks, indicating that MyD88-GFP is recruited to the MyD88 mCherry seeds (Fig. 3b). Moreover, with time, the events detected in the GFP channel became brighter than those in the Cherry channel, indicating that the GFP-tagged MyD88 grow off the mCherry-tagged MyD88 seeds.

Figure 3c shows that seeding of MyD88 polymerisation occurs over a large range of concentrations. This is particularly obvious within the range of sub-threshold concentrations, where polymerisation does not normally take place on the timescale of our experiment. Ultimately, this allowed us to define the critical concentration for the polymerisation of FL MyD88. Below this critical concentration (10 nM), full-length MyD88 does not polymerise, even in the presence of seeds. In the supercritical zone (> 120 nM), full-length MyD88 can spontaneously polymerise, but addition of seeds increases the effect and the plateau in brightness values is reached earlier. A large metastable zone (10–120 nM) exists, where the tendency of MyD88 to polymerise on its own is low in the timescale of our experiment, but can be catalysed by the presence of the polymeric seeds.

Biologically, the existence of this metastable zone is important, as it shows that rapid amplification of MyD88 signalling can be achieved through seeding. The “in vitro” seeding is the introduction of MyD88 filaments; however, in vivo, seeding can be triggered by upstream proteins such as the recruitment of MyD88 through Mal nucleation. The depth of the metastable zone is also important: if this zone is too narrow, the system would respond too fast, initiating the highly effective pro-inflammatory innate immune response prematurely. A large metastable zone is therefore more physiologically desirable [30].

### Disease-associated point mutations abrogate MyD88s ability to optimally polymerise

Having established that full-length MyD88 can undergo an active polymerisation process, we then investigated whether pathological point mutations could affect this protein polymerisation propensity. Hence, the L93P, R196C and L252P point mutations were individually introduced into the GFP-tagged full-length MyD88. Once again, expression of tagged MyD88 by the cell-free translation system was used and fluorescence time-traces were measured and plotted as distributions of fluorescence intensities.

**Fig. 4:**
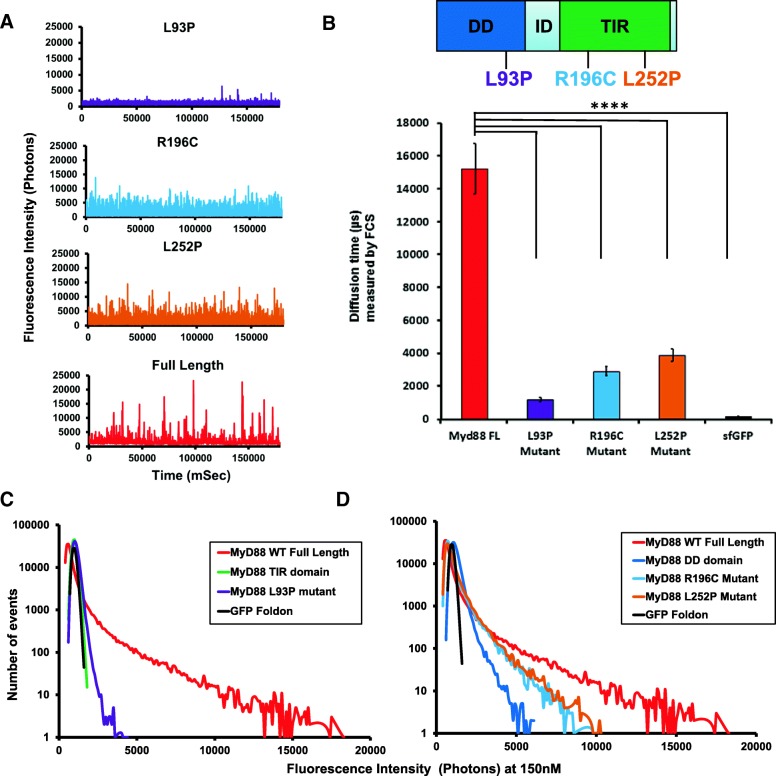
Disease-associated point mutations abrogate domain function and thus, MyD88 polymerisation. **a** Fluorescence time-traces obtained with disease-associated point mutants of the full-length MyD88 protein, as well as the wild-type full-length MyD88 at a protein concentration of 150 nM. As in Fig. 1, the diffusion of an oligomer equates to the same number of fluorophores moving through the confocal volume, creating a burst of fluorescence in the time-trace being directly proportional to the size of the oligomer. **b** Diffusion time (μs) measured by FCS showing the drastic shift in diffusion time when comparing the mutants to the wild-type protein. **c** Fluorescence intensity histogram showing that the L93P point mutation, which is within the DD, in GFP-tagged MyD88 renders the polymerisation propensity similar to the MyD88 TIR domain alone. **d** Fluorescence intensity histogram demonstrating that the R196C and L252P point mutations (present within the TIR domain) in the GFP-tagged MyD88 render the polymerisation propensity more similar to the MyD88 DD alone. Fluorescence time-traces and intensity histograms in **a**, **c** and **d** are representative of eight independent experiments. Values in **b** are ±SD from these eight measurements. Sidak’s multiple comparisons test (****P < 0.0001)

In Fig. 4a, typical fluorescence time-traces obtained when all proteins were expressed at 150 nM concentrations reveal a different profile for the mutants, compared to the wild-type (WT) protein, with a loss of the brighter objects for all mutants. This is confirmed by the FCS data that demonstrate a reduction in sizes of the larger protein species, compared to the WT protein (Fig. 4b). Brightness profiles of the full-length MyD88 mutants were compared to those obtained for the isolated domains (Fig. 4c, d, e). The full-length MyD88 L93P mutant polymerisation profile roughly mimics that of a MyD88 TIR domain alone, while the full-length R196C and L252P mutant polymerisation profiles show a behaviour in between those of MyD88 DD alone and full-length MyD88. This suggests that the two point mutants have a higher tendency to oligomerise than the isolated DD, but they do not support the formation of the higher order assemblies observed with the WT protein. Overall, it appears that the point mutations decrease the capacity of the domains to contribute to polymerisation, possibly through impairing homotypic protein-protein interactions (PPIs).

### L252P mutants form stable oligomers at a 40-fold lower concentration than wild-type MyD88

**Fig. 5:**
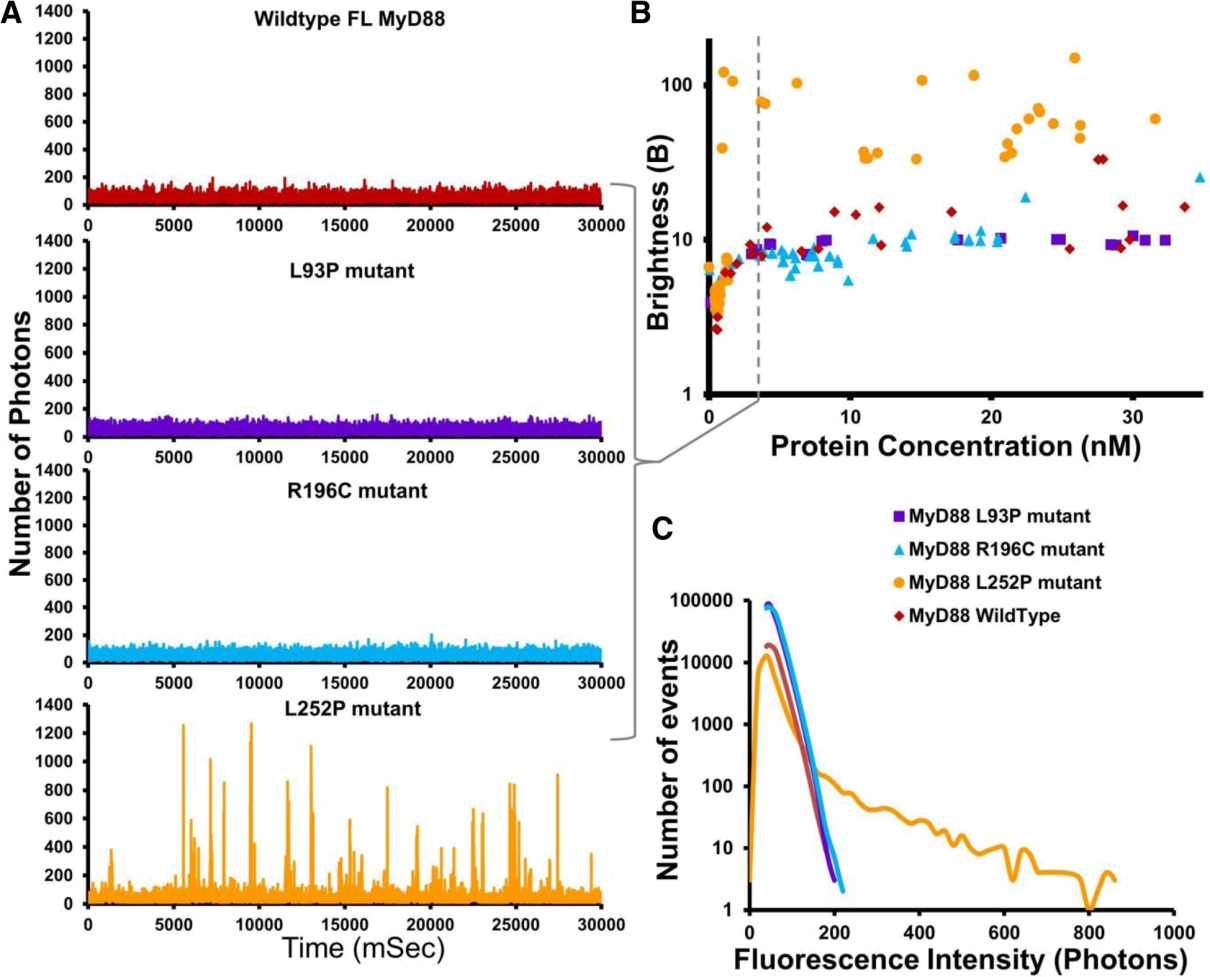
Mutations in same domain lead to contrasting disease phenotypes; cancer-causing L252P mutation lowers threshold for MyD88 oligomerisation. **a** Fluorescence time-traces obtained at 3 nM protein concentration of the disease-associated point mutants in the full-length MyD88 protein, as well as full-length wild-type MyD88, demonstrating the stability of the L252P point mutant. **b** The *B* parameter (brightness) correlates with the number of oligomers detected in typical time-traces as a function of protein concentration (nM). **c** Fluorescence intensity histogram demonstrating the stable L252P oligomer still forming at 3 nM, in comparison to the other constructs. Fluorescence time-traces in **a** are representative traces obtained at 3 nM protein concentrations, **c** is the representative fluorescence intensity histogram. Values in **b** are from approx. 60 dilution experiments with the various protein concentrations and corresponding brightness values obtained plotted. Fluorescence intensity values at 3 nM are statistically significant with *P* < 0.0001 between L252P mutant values and the other mutants

We then examined the behaviour of the mutants as a function of protein expression, taking advantage of the control one can exert with the cell-free translation system. Figure 5a shows the differences in polymerisation profiles exhibited by the mutants at the same low 3 nM concentrations. This contrasts with the profiles in Fig. 4, obtained at 150 nM. At this low concentration, we do not detect the presence of large objects for WT MyD88 or for any of the mutants and the traces obtained for WT, L93P and R196C MyD88 suggest the presence of mainly monomeric species. In contrast, L252P still seems able to oligomerise, as indicated by the presence of fluorescence bursts. To confirm this unexpected effect, the oligomerisation thresholds of the mutant MyD88 proteins were measured and analysed by plotting the *B* parameter as a function of protein concentration (Fig. 5b). In the case of R196C and L93P, the *B* values never reach those of the wild-type protein, indicating that the pathological point mutants alone cannot propagate polymerisation, no matter what concentration of protein is achieved (within the range of our experiment). The L252P mutant also never formed the large aggregates that are observed with WT MyD88 when expressed within our system. Strikingly though, at very low concentrations, where WT MyD88 and the other disease-associated point mutants exist only as monomers, the L252P mutant still forms stable low-order oligomers (Fig. 5b). The threshold for oligomerisation is extremely low (approximately 2 nM), in the subcritical zone of WT MyD88. Interestingly, this threshold concentration correlates with the concentration above which WT MyD88 can be caused to polymerise by seeding (Fig. 3c), suggesting that the L252P oligomers could be acting as an activated form of MyD88 [7].

The presence of L252P oligomers had been postulated previously based on from computational model studies [31], which predicted the existence of these oligomers at levels that are physiologically present in inactivated cells, i.e. without expression upregulation upon receptor-ligand binding and activation. This fits well with our observations and our data confirm the existence of these extremely stable low-order oligomers of MyD88.

### Mutations within the same domain can lead to contrasting protein properties

**Fig. 6:**
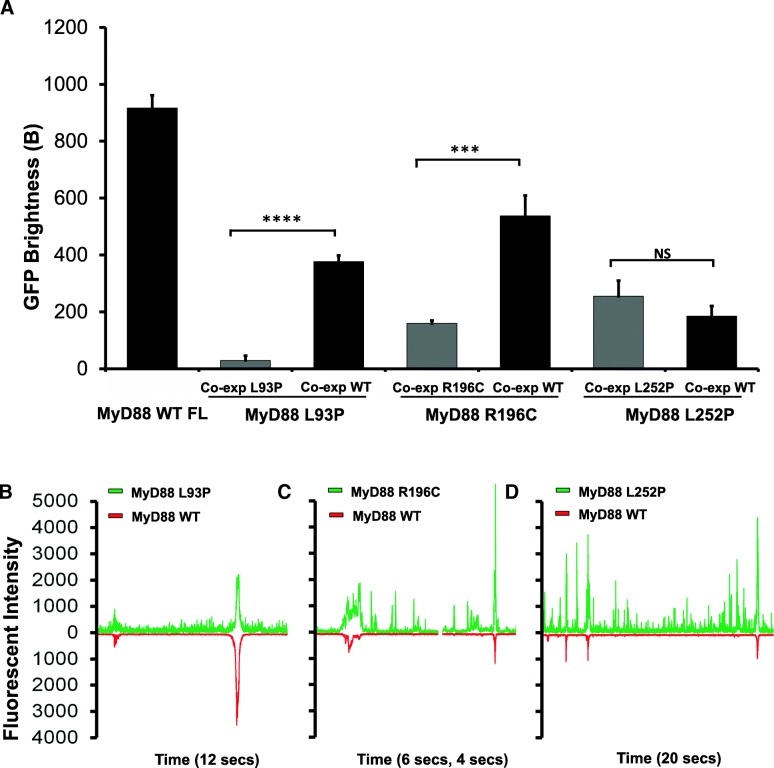
Co-expression with wild-type full-length MyD88 partially rescues the ability of the recurrent bacterial infection disease-associated point mutants to polymerise. **a** GFP brightness histogram of the mCherry-tagged wild-type MyD88 co-expressed with disease-associated mutants (simulating heterozygous expression in patients), as well as L93P, R196C or L252P mutant proteins co-expressed with themselves (i.e. homozygous protein expression) and wild-type MyD88 alone as a control. GFP brightness from mutants and WT measured. **b–d** Fluorescence time-traces of the disease-associated mutants co-expressed with mCherry-tagged WT MyD88. The recurrent bacterial infection disease-associated point mutation, L93P (**b**) and R196C (**c**), co-expression rescue experiments contrast with the continuously oligomerising L252P mutant whereby (**d**) L252P is not rescued and exists as its own separate population. Values are mean ± SD from six independent experiments (**a**) with representative traces from these experiments shown in (**b–d**). NS > 0.9999, ***P = 0.0001, ****P < 0.0001

Our data also show a drastic difference in the behaviour between L252P and R196C mutants, even though both residues are located within the same TIR domain. Differences in oligomerisation pattern could potentially explain the differences in the related pathologies, with the L252P protein producing stable oligomerisation at low concentrations leading to cancer, while the R196C protein producing a lack of oligomerisation/polymerisation propensity, leading to a dampening of the innate immune response to bacterial infection. However, these are not the only differences uncovered between these disease-associated mutants. The L252P mutation is a dominant mutation, whereas L93P and R196C are both recessive mutations. Because primary immunodeficiency only affects homozygous or compound heterozygous carriers of the point mutations L93P and R196C, we hypothesised that polymeric propagation could be rescued by the presence of the wild-type protein. To test this, GFP-tagged mutants and mCherry-tagged WT MyD88 were co-expressed in LTE and subjected to our brightness assay. The brightness parameters for the mutants obtained through single or co-expression could then be compared (Fig. 6a). In the case of L93P and R196C, the GFP brightness value is significantly larger upon co-expression, indicating that higher order polymers of the mutant MyD88, when expressed with WT FL mCherry MyD88, were forming. Indeed, examination of the fluorescence time-traces reveals the presence of coincident peaks (Fig. 6b–d), showing that WT MyD88 can recruit the mutants into its polymers. The overall degree of polymerisation is still lower than in the case of the wild-type protein alone, but the ability of the system to form large objects may be sufficient to restore normal signalling.

In contrast, the brightness of L252P upon co-expression is unchanged (Fig. 6a), indicating that the mutant species oligomerises regardless of whether the wild-type protein is present. Furthermore, few coincident peaks were detected (Fig. 6d), showing that WT MyD88 does not recruit this mutant into its polymers as readily as L93P and R196C (Fig. 6b, c). The differential incorporation of the mutants into the wild-type polymers correlates well with what is observed at the physiological level. Heterozygous patients carrying the L93P or R196C mutations do not suffer from the recurrent bacterial infections. The wild-type protein polymerisation, as well as the incorporation of the mutants into the polymerising wild-type protein, albeit at suboptimal levels (Fig. 6a), may be sufficient to propagate signalling efficiently. Impairment of polymerisation and subsequent signalling is only observed in the absence of wild-type MyD88, as would be the case for homozygous and compound heterozygous carriers (i.e. both alleles of the gene harbour mutations such as L93P and R196C) [4, 5]. In the case of L252P, a distinct population of finite-sized oligomers seems to always exist, irrespective of the presence of WT MyD88 (Fig. 6a, d). This would correlate with the fact that both the heterozygous and homozygous patients suffer from associated cancers [32].

### L252P can seed WT MyD88 and recruit IRAK4

**Fig. 7:**
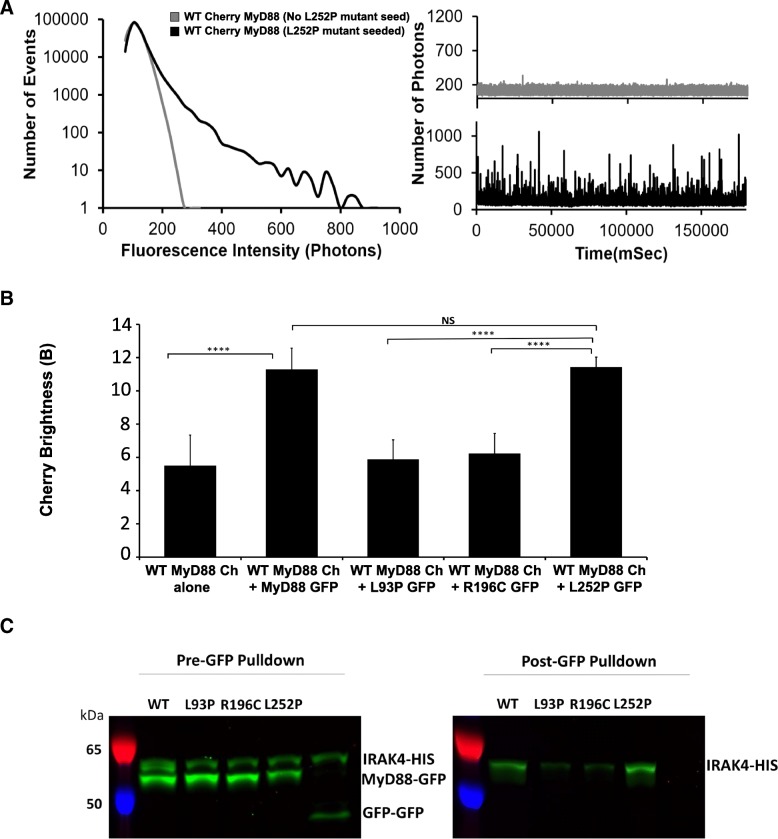
Gain of function point mutation, L252P, recruits both wildtype MyD88 and downstream IRAK4. **a** Example fluorescence intensity histogram showing the impact of the L252P point mutation on WT FL MyD88. Corresponding fluorescence time-traces obtained for full-length mCherry MyD88 protein at subcritical concentration (5 nM) and then with the addition of full-length L252P mutant GFP seed. The diffusion of the Cherry MyD88 protein equates to the same number of fluorophores moving through the confocal volume, creating a burst of fluorescence in the time-trace being directly proportional to the size of the oligomer. **b** Brightness histogram of the mCherry-tagged wild-type MyD88 (expressed at subcritical concentration) co-expressed with disease-associated mutants (simulating heterozygous expression in patients), as well as L93P, R196C or L252P mutant proteins co-expressed with themselves (i.e. homozygous protein expression) and wild-type MyD88 alone as a control. mCherry brightness from WT MyD88 measured in three independent experiments. NS > 0.9999, ***P = 0.0001, ****P < 0.0001. **c** GFP pulldown of GFP-tagged MyD88 WT, L93P, R196C or L252P mutant proteins co-expressed with IRAK4-HIS tagged. IRAK4-HIS tagged 58 kDa, MyD88-GFP tagged 53.2 kDa, GFP dimer control 40 kDa. Pre-pulldown and post-pulldown shown for MyD88 WT, L93P, R196C, L252P mutant and GFP dimer control. GFP not visible in post-pulldown due to boiling step. HIS-tagged IRAK4 labelled with bodypi. Example gel from three independent experimental repeats

To test whether small oligomers of L252P could serve as seeding events for WT MyD88, we again used our seeding assay. Here, WT Myd88 full-length tagged with mCherry was expressed as a monomeric protein (Fig. 7a, grey traces). Upon addition of separately expressed GFP-L252P, peaks are detected in the red channel (Fig. 7a, black traces) indicating that WT MyD88 is now self-associating. As shown in Fig. 7b, only L252P, and not L93P or R196C, is able to induce an increase in WT MyD88 brightness and therefore induce MyD88 polymerisation.

An important question about the L252P oligomers is whether they are signalling competent. In vivo and cell data support this hypothesis. Studies have demonstrated that IRAK4 inhibition promoted killing of ABC DLBCL lines harbouring MyD88 L252P, by down-modulating survival signals, including NF-κB [33]. The established link between the L252P mutation and the occurrence of cancer allows us to hypothesise that the stable oligomers formed by this mutant may be all that is required for constitutive signalling.

In our system, we tested the ability of the L252P mutant to recruit IRAK4, as a proxy for its ability to signal. GFP-tagged WT MyD88 and mutants was added to a solution of His-tagged IRAK4. IRAK4 was fluorescently labelled during synthesis in the LTE system by addition of bodypi-lysines. GFP-nanotrap presenting sepharose beads were used to immuno-precipitate the GFP-tagged MyD88 constructs. The beads-bound fraction were then treated at 95 °C to release the proteins from the GFP-nanotraps. This treatment results in unfolding of GFP and loss of fluorescence of the MyD88 constructs, but does not affect the fluorescence of the bodypi-labelled IRAK4. Therefore, the amount of the IRAK4 that has been co-immunoprecipitated can be detected easily on a SDS-page gel scanned for fluorescence. This experiment shows that L252P is capable of recruiting IRAK4 to the same extent as the WT MyD88. On the contrary, L93P and R196C have a reduced capacity to recruit IRAK4 compared to WT MyD88. This supports the idea that L252P could act as an activator of MyD88. Further validation in vivo will be required to fully characterise the mechanisms leading to enhanced NFkB signalling observed in previous studies [34].

## Discussion

### Full-length MyD88 biophysical behaviour

Here, we studied the contribution of the domains and the effect of physiological mutations on the biochemical and biophysical behaviour, in particular on the polymerisation propensity of MyD88, a key protein in TLR pathways. To characterise the formation of protein assemblies, we utilise single molecular fluorescence spectroscopy, as this technique has the unique ability to quantify oligomers and track conformational changes at a single protein level. Through utilising cell-free eukaryotic expression, we can co-express proteins together in their known complexes, allowing the native and physiological PPIs to occur. We can also control expression and, therefore, distinguish thresholds, aggregation propensity, and self-propagating behaviour.

When we first compared the isolated domains with the full-length protein, we demonstrated that only full-length MyD88 is capable of forming large objects in a concentration-dependent, self-templated manner. Traditionally, the biochemical studies of MyD88 and other adaptors have mainly focused on the role of the isolated domains, partly due to the difficulty of purifying the full-length proteins. From the study of these isolated domains, two mechanisms of self-assembly have been described. For many years, TIR domain associations, which are weak and transient, have been seen as contributing little to the oligomerisation status of signalling proteins [35, 36]. However, the recent cryoEM structure of Mal TIR domain in filamentous form [12], as well as single-molecule imaging in live macrophages [26], demonstrated that upon specific ligand binding, TIR-containing proteins cooperatively assemble into large multi-protein complexes [12, 29]. The TIR domain of MyD88 was also demonstrated to polymerise but only upon seeding by Mal filaments. On the other hand, the DD has been known to participate in the formation of the higher order helical assembly of the myddosome, a signalling complex that also includes the DDs of IRAK2 and IRAK4. Our data using the isolated domains recapitulate those findings (Fig. 2). We show that the TIR domains alone are present as small, low-order oligomers, no matter what concentration of protein is expressed and that these TIR domain oligomers never combine to form large signalosomes on their own (Figs. 1 and 2). In our system, MyD88 DD is able to form well-defined oligomers, consistent with previous results [11]. Interestingly, the DD appears to exhibit concentration-dependent “all-or-none” monomeric to oligomeric behaviour, albeit at a much lower scale than full-length MyD88 (Fig. 1b inset). This threshold is potentially driving the assembly of the Myddosome, with the mutants of the DD affecting its formation and function. The behaviour of the isolated domains is in stark contrast to the full-length protein, which has a tendency to form large complexes even at these low concentrations (Fig. 1). Both the TIR and death domains can drive oligomerisation, but the combination of the two domains is required for efficient polymerisation. Our seeding assay also reveals a large zone of concentrations where the full-length protein is metastable. We hypothesise that the presence of the two domains contributes to the creation of this metastable zone. Having two domains has been demonstrated to create auto-inhibition within full-length proteins so as to hinder spontaneous assembly. Comparing spontaneous aggregation of full-length MyD88 with the isolated domains reveal that monomeric full-length is more stable, with a characteristic critical concentration for aggregation (*C*_c_) around 150 nM, compared to *C*_c_^TIR^ ≈ *C*_c_^DD^ ≈ 50 nM. This seems to indicate that the presence of the two domains creates an auto-inhibitory system that is less prone to self-activation.

### Pathological point mutations lead to both loss and gain of function

Previous studies have uncovered the impact of disease-associated point mutations of MyD88 in regard to the heterotypic protein-protein interactions that occur with the other components of the signalling pathway [4, 37, 38]. Here, we characterised the polymerisation propensity of three pathological mutants in comparison to wild-type full-length MyD88. All point mutants showed a reduced ability to form polymers compared to the WT protein. However, studying the concentration-dependence of the aggregation process reveals striking differences. Both L93P and R196C have an increased *C*_c_ for self-assembly compared to the wild-type, indicating a loss of function. In contrast, the *C*_c_ for L252P is greatly decreased and self-assembly occurs at much lower concentration, pointing at a gain-of-function mutation.

Recently, Ve et al. demonstrated that the R196 mutant completely abolished Mal TIR-induced assemblies of MyD88 TIR, as well as the ability of full-length MyD88 to cluster in HEK293 cells. We wished to uncover the impact of the R196C mutation alone in regard to the homotypic interactions that underlie the self-association of full-length MyD88. Our data demonstrates that a R196C point mutation alone reduces MyD88’s ability to homotypically interact and polymerise as seen with the wild-type protein. The R196C mutant has also been found to have reduced PPIs with other TIR domain-containing signalling proteins [4], further reducing its ability to signal.

The L93P mutation is localised in the death domain (DD). As the highly conserved L93 side chain is buried, the L93P mutation, as well as affecting the helix formation, would disrupt the hydrophobic core of the DD [9]. The view is that this point mutation renders the DD non-functional and prevents optimal binding to upstream signalling proteins, as well as fully abrogating binding to downstream signalling proteins, such as the kinases like IRAK4 [4] that propagate the signal. This has been proven in regard to the myddosome (“DD” alone complex) and our pull down data of the full-length MyD88 WT and mutants with IRAK4 is consistent with this (Fig. 7). In our system, L93P is unable to form polymers to the same extent as wild-type MyD88 and behaves similarly to the isolated TIR domain. Although L93P and R196C occur in the two different domains of MyD88, one in the DD and the other in the TIR domain, they both cause autosomal recessive MyD88 deficiency that results in life-threatening, recurrent pyogenic bacterial infections. We show that at the molecular level the two proteins behave similarly, as they both exhibit a reduced ability to polymerise and can both be partially incorporated (“rescued”) by the presence of the wild-type protein. This may explain the recessive character of the disease, as only homozygous or compound heterozygous carriers display the disease phenotype.

On the other hand, it is intriguing that although both R196C and L252P occur in the same TIR domain, one gives rise to recurrent bacterial infections associated with the immunodeficiency, while the other results in lymphoma. So far, computational methods have been used to characterise the conformational effects of the L252P mutation [7, 34, 39]. Molecular dynamics simulations revealed that the L252P mutation allosterically quenched the global conformational dynamics of the TIR domain and readjusted its salt bridges and dynamic community network. The dampened motion restricts its ability to heterodimerise with other TIR domains, thereby curtailing physiological signalling. Interestingly, the mutation was also predicted to enhance signalling by stabilising the core of the homodimer interface of the MyD88-TIR domain [31]. It must be noted that these models were established before the elucidation of the structure of the filament. Our experimental results show, for the first time, that L252P forms extremely stable oligomers compared to the wild-type protein as well as to the other mutants we have studied. We could observe oligomers when the protein was expressed at concentrations as low as approximately 3 nM. As suggested, the conformational dynamics of cancer-associated MyD88-TIR domain mutant L252P seem to allosterically tilt the landscape toward homo-oligomerisation in vitro, which would propagate a signal independent of the TLR receptor activation [34].

## Conclusions

Our observations that pathological mutations have profound effects on self-assembly illustrates that prion-like polymerisation is a fundamental mechanism of intracellular communication within the innate immune system. Development of drugs that can interfere with the higher order oligomerisation and polymerisation of adaptor proteins would therefore be a novel advancement in medicine, with the potential to function as anti-inflammatory as well as anticancer agents. The MyD88 L252P (also referred to as L265P) mutation is implicated in almost 100% of Waldenstrom’s macroglobulinemia (WM) cases, 2–10% of chronic lymphocytic leukaemia (CLL) cases, 69% of cutaneous diffuse large B cell lymphoma (DLBCL) cases, and 38% of primary central nervous system lymphoma (PCNSL) cases [39–41]. The activated B cell-like (ABC) subtype of diffuse large B cell lymphoma (DLBCL) remains the least curable form of this malignancy, with less than a 40% cure rate [42]. L252P mutation in MyD88 was identified in tumour samples from 49 of 54 patients with the incurable form of the disease. Since 2016, testing for MyD88 was added to the essential recommendations for initial work-up of lymphoplasmacytic lymphoma/Waldenstrom’s macroglobulinemia (LPL/WM) in the National Comprehensive Cancer Network (NCCN) Guidelines. Based on the strong link between the mutation and cancer, we can hypothesise that the increase in oligomerisation we observe has a physiological impact. Therefore, the stable signalling oligomers created by MyD88 L252P would be an enticing target from a therapeutic standpoint.

## Materials and methods

### Preparation of LTE

Cell-free lysate was collected from *Leishmania tarentolae* (*LT*) as per Johnston & Alexandrov [22, 43, 44]. *Leishmania tarentolae* Parrot strain was acquired as LEXSY host P10 from Jena Bioscience GmbH, Jena, Germany, and cultured in the TBGG medium containing 0.2% *v*/*v* penicillin/streptomycin (Life Technologies) and 0.05% *w*/*v* hemin (MP Biomedical). LT cells were harvested through centrifugation at 2500×*g*, washed twice by resuspension in 45 mM HEPES (pH 7.6) containing 3 mM magnesium acetate, 100 mM potassium acetate, and 250 mM sucrose. Cells were resuspended to 0.25 g cells/g suspension and incubated under 7000 kPa nitrogen for 45 min, then lysed by rapid release of pressure in a cell disruption vessel (Parr Instruments, USA). Through sequential centrifugation at 10,000×*g* and 30,000×*g*, the cell-free lysate was clarified and 10 μM anti-splice leader DNA leader oligonucleotide was added. The cell-free lysate was then desalted into 45 mM HEPES (pH 7.6) containing 100 mM potassium acetate and 3 mM magnesium acetate. The LTE was supplemented with a coupled translation/transcription feeding solution and snap-frozen until required for further experimentation.

### Gateway plasmids for cell-free protein expression

Full-length MyD88, MyD88 TIR domain (amino acids 159–296), MyD88 DD (amino acids 1–117), and all of the other proteins from the pathway were cloned into the Gateway destination vectors: N-terminal GFP-tagged (pCellFree_G03), N-terminal mCherry-tagged (pCellFree_G05), C-terminal eGFP-tagged (pCellFree_G04) or C-terminal mCherry-cMyc-tagged (pCellFree_G08), facilitating cell-free expression [45]. The Gateway PCR cloning protocol was used and entry clones were generated with PCR primers to attB1 and attB2 sites (forward primer: 5′GGGGACAAGTTTGTACAAAAAAGCAGGCTT (nnn)_18–25_ 3′, reverse primer: 5′GGGGACCACTTTGTACAAGAAAGCTGGGTT (nnnn)_18–25_ 3′) [46].

### Cloning point mutations

Primers were designed and ordered through IDT. Cloning was conducted as per Phusion® High-Fidelity DNA Polymerase protocol with full-length MyD88 N-terminal GFP tagged (pCellFree_G03) and C-terminal mCherry-cMyc tagged (pCellFree_G08) as donor construct. All mutant sequences were verified by the Ramaciotti UNSW Sequencing Facility.

**Table:** 

MyD88 Mutant L93P Forward	GTAGGCCGACTGCTCGAGCTGCCTACCAAGCTGGGCCGCGAC
MyD88 Mutant L93P Reverse	AGAGGCGCCAGGGCGTCCCTGCCA
MyD88 Mutant R196C Forward	CGACTGAAGTTGTGTGTGTCTGACTGCGATGTCCTGCCTGGCACC
MyD88 Mutant R196C Reverse	ATAGTTTGTCTGTTCCAGTTGCCGGAT
MyD88 Mutant L265P Forward	GGTGCCCATCAGAAGCGACCAATCCCCATCAAGTACAAGGCAATG
MyD88 Mutant L265P Reverse	TGGAGAGAGGCTGAGTGCAAATTT

### In vitro protein expression

All proteins were expressed through the addition of LTE lysate to DNA template (in a ratio of 1:9) for 2.5 h at 27 °C and 0.5 h at 37 °C. For protein dilution titrations, the concentration of DNA template was varied using serial dilutions with nuclease-free H_2_O covering ranges from 600 nM stock to 50 nM concentrations correlating with protein concentrations of 300 to 0 nM. One microlitre of the diluted DNA was used to prime 9 μL of LTE. Samples were processed immediately for either fluorescence microscopy analysis, seeding experiments, or AlphaScreen assay.

### Single-molecule fluorescence spectroscopy

Single-molecule spectroscopy was performed as described in previous studies by Sierecki et al. and Gambin et al. [23, 26]. The proteins were labelled with genetically encoded fluorophores (GFP and mCherry) facilitating fluorescence spectroscopy under a confocal microscope directly in the cell-free expression mixtures, without any purification steps. Two overlapping lasers excite the GFP and mCherry fluorophores, creating a small detection volume in which GFP and mCherry fluorescence emitted by proteins is recorded on single-photon-counting detectors. Due to Brownian motion, the proteins freely diffuse, constantly entering and exiting the detection volume of the microscope and creating fluctuations in the fluorescence intensity. The number of photons collected versus the time of the measurements is obtained as raw data. Then the amplitude and frequency of the fluorescence fluctuations are quantified to characterise the oligomerisation status of the proteins [47].

N-terminal trimeric foldon and GFP-foldon (both known to be trimeric proteins and used as a known size control) were expressed for quantification of the intensity measurements. A 488-nm laser beam was focused in the sample volume using a 40×/1.2 NA water immersion objective (Zeiss). The fluorescence of eGFP was measured through a 525/20-nm band pass filter, and the number of photons collected in 1 ms time bins (*I*(*t*)) was recorded. The proteins were diluted 10 times in buffer A.

The fluorescent time-trace *I*(*t*) obtained shows the presence of intense bursts of fluorescence, with values well over the typical fluctuations of *I*(*t*). The presence of these bursts increases the standard deviation of the distribution. To compare the aggregation at different concentrations, we used the *B* parameter, this being independent of the protein concentration and can be written as:

$$ B=\frac{{\left(\mathrm{Standard}\ \mathrm{deviation}\ (I)\right)}^2}{\mathrm{average}\ (I)} $$

### Single-molecule fluorescence spectroscopy: seeding experiments

In this assay, full-length MyD88 was expressed as an mCherry-tagged protein. The aggregates were spun down and sonicated, and then added to a solution of monomeric GFP protein. We directly detected the recruitment of the GFP monomer to the seed with two-colour coincidence measurement, by detecting the simultaneous presence of a signal in the GFP (green) and mCherry (red) channels. mCherry-tagged seeds of full-length MyD88 were expressed in LTE by the addition of the template DNA in 10 μL lysate, generating a final concentration of ~ 30 nM protein. To produce the seeds, the samples were then spun down at 13,000×*g* for 5 min. Eighty percent of the supernatant was discarded, and the solution was sonicated for 1 min in a water bath. During sonication, GFP-tagged full-length MyD88 was expressed, as previously described using serial dilutions to generate a range of GFP-tagged protein concentrations from ~ 25 to 0 nM and diluted ten times before being placed under the microscope. Two lasers (488 nm and 561 nm) were focused in solution with a 40×/1.2-NA water-immersion objective (Zeiss). Fluorescence was collected and separated with a 565-nm dichroic mirror; fluorescence from GFP was passed through a 525/20-nm band-pass filter, and fluorescence from mCherry was filtered by a 580-nm long-pass filter. The fluorescence of the two channels was recorded simultaneously in 1-ms time bins. Fluorescence time-traces were recorded for 180 s.

### Single-molecule fluorescence spectroscopy: co-expression experiments

MyD88 point mutations with N-terminal-tagged eGFP were co-expressed with full-length MyD88 C-terminally tagged with mCherry-cMyc in the respective ratios of 20 and 40 nM of DNA template, in 10 μL of LTE for 2.5 h at 27 °C and 0.5 h at 37 °C, then measured on the microscope and analysed as per all single-molecule spectroscopy experiments described above.

### GFP pulldown

MyD88 WT and point mutations with N-terminal-tagged eGFP were expressed as described above. IRAK4 FL-HIS tagged (labelled with bodypi) was expressed separately. After 2.5 h expression, MyD88 proteins and IRAK4 protein samples were mixed at a ratio of 1:1 and incubated at 27 °C for 30 min. NaCl was added to a final concentration of 200 mM. Ten microlitres of GFP nanotrap beads was added to a MyD88-IRAK4 sample of 50 μL and incubated at room temperature for 15–20 min (while shaking). The sample was spun down at 1000 rpm for 2 min. Supernatant was removed and beads were washed six times (Add 200 μL of 1× PBS, resuspend beads, spin down: 1000 rpm for 2 min, remove wash solution from above beads). Before addition to gel, 1× LDS was added and samples were boiled at 95 °C for 5 min with GFP denatured but bodypi (IRAK4-HIS) remaining visible on the gel.

### Experimental design

For all experiments represented in Figs. 1, 2, 3, 4, 5 and 6, samples were repeatedly expressed in aliquots from the same batch of LTE lysate. Repeats were carried out on different days and within different samples of the expression lysates. The extent of variation found was reported in the standard deviations. *P* values were obtained following ANOVA using the Holm-Sidak multiple comparison test. As the values represent the size of the oligomers and the impact of domains and mutations, this was appropriate to assess the significance of these values when compared.

## Abbreviations

ASC: Apoptosis-associated speck-like protein containing a CARD
B: Brightness
CARD: Caspase recruitment domain
DD: Death domain
FCS: Fluorescence correlation spectroscopy
FL: Full-length
GFP: Green fluorescent protein
ID: Intermediate domain
IRAK: Interleukin-1 receptor-associated kinase
LTE: *Leishmania tarentolae* extracts
MAVS: Mitochondrial antiviral-signalling protein
MyD88: Myeloid differentiation primary response 88
PCH: Photon counting histogram
PPI: Protein-protein interaction
PRR: Pattern recognition receptor
PYD: Pyrin domain
TIR domain: Toll/interleukin-1 receptor domain
TLR: Toll-like receptor
WT: Wild-type
